# Identifying the Gene Signatures from Gene-Pathway Bipartite Network Guarantees the Robust Model Performance on Predicting the Cancer Prognosis

**DOI:** 10.1155/2014/424509

**Published:** 2014-07-14

**Authors:** Li He, Yuelong Wang, Yongning Yang, Liqiu Huang, Zhining Wen

**Affiliations:** ^1^Biogas Institute of Ministry of Agriculture, Chengdu 610041, China; ^2^College of Chemistry, Sichuan University, Chengdu 610064, China

## Abstract

For the purpose of improving the prediction of cancer prognosis in the clinical researches, various algorithms have been developed to construct the predictive models with the gene signatures detected by DNA microarrays. Due to the heterogeneity of the clinical samples, the list of differentially expressed genes (DEGs) generated by the statistical methods or the machine learning algorithms often involves a number of false positive genes, which are not associated with the phenotypic differences between the compared clinical conditions, and subsequently impacts the reliability of the predictive models. In this study, we proposed a strategy, which combined the statistical algorithm with the gene-pathway bipartite networks, to generate the reliable lists of cancer-related DEGs and constructed the models by using support vector machine for predicting the prognosis of three types of cancers, namely, breast cancer, acute myeloma leukemia, and glioblastoma. Our results demonstrated that, combined with the gene-pathway bipartite networks, our proposed strategy can efficiently generate the reliable cancer-related DEG lists for constructing the predictive models. In addition, the model performance in the swap analysis was similar to that in the original analysis, indicating the robustness of the models in predicting the cancer outcomes.

## 1. Introduction

In the past decade, DNA microarray technology has been widely used in clinical researches to predict the cancer outcomes because of its capability of monitoring tens of thousands of genes simultaneously [1–9]. Accurately identifying the genes, for which the changes of their expression levels are significantly correlated with the phenotypic differences between the clinical conditions, plays an important role in the procedure of clinical model construction. The statistical methods and the machine learning algorithms that are routinely used for gene selection mainly identify the differentially expressed genes (DEGs) according to the changes of the gene expression levels between the compared biological samples. However, because of the heterogeneity of the clinical samples, the changes of the gene expression levels may be not only caused by the changes in the status of the cancer cells but also by those of the cells unrelated to the cancers. In addition, the intensities detected by the microarrays for a gene will vary to some extent among the technical replicates due to the complex procedures of the microarray experiment, such as labeling, hybridization, and scanning. Consequently, the DEG list generated by the statistical methods or the machine learning algorithms often involves a number of false positive genes, which are not associated with the phenotypic differences between the compared clinical samples, and subsequently impacts the reliability of the predictive models.

The network-based methodologies can efficiently integrate the biological information with the computational techniques and link the disease-related genes to relevant proteins and disease types. In recent years, the network-based methodologies have been successfully introduced into the systems biology researches for drug discovering [2], identifying disease-related genes [10–15], and revealing the molecular mechanisms of tumorigenesis [16–19]. In clinical researches, the prediction models constructed with the cancer-related gene markers, which were selected only by the statistical methods or the machine learning algorithms, cannot ensure the accuracy and the reproducibility in predicting the clinical outcomes of cancer patients. Therefore, for the sake of improving the model performance and interpreting the biological relevance of the gene markers and the specific cancer, a number of network-based algorithms were developed to prioritize the prognostic genes [20–38].

In our study, a new strategy, which combined the statistical algorithm with a gene-pathway bipartite network, was proposed to prioritize the reliable gene signatures and the supported vector machine (SVM) was used to construct the models for predicting the clinical outcomes of cancer patients. The DEG list was firstly generated by the statistical methods, for example, Student's *t*-test. Then, the bipartite network that connected the genes and the cancer-related pathways was constructed to score each of the DEGs according to its connectivity in the network. Finally, the DEGs were ranked by the scores in descending order and those, for which the scores were greater than a given cutoff, were selected as features for predicting the cancer prognosis.

To evaluate the performance of the predictive models with the gene signatures generated by our strategy, three data sets including the gene expression data of the clinical samples collected from the patients of breast cancer, acute myeloma leukemia, and glioblastoma were downloaded from the gene expression omnibus (GEO) database. Gene signatures separately identified from these data sets by our strategy were used as features to predict the reoperative treatment response of breast cancer, the overall survival milestone outcome of acute myeloma leukemia, and the molecular subclasses of high-grade glioblastoma. The results of predicting the reoperative treatment response of breast cancer and the overall survival milestone outcome of acute myeloma leukemia showed that our models performed better than those reported by the data contributors. In addition, the accuracy of predicting the molecular subclasses of high-grade glioblastoma was as high as 87.5%. In the swap analysis, we repeated the model construction and validation process by training the models with the original independent test set and validating them using the original training set with the same gene signatures prioritized in the original analysis. The prediction results were similar to those achieved in original analysis, indicating the robust model performance on predicting the cancer prognosis when using the gene signatures identified by our proposed strategy.

## 2. Materials and Methods

### 2.1. Data Sets

All microarray gene expression data (series MATRIX files) generated from the clinical samples of breast cancer, acute myeloma leukemia, and glioblastoma and the corresponding clinical information were downloaded from the National Center for Biotechnology Information's Gene Expression Omnibus (GEO) database (series accession numbers: GSE16716, GSE12417, and GSE13041).

In the human breast cancer data set [4, 9], the gene expression data of 230 clinical samples were generated by using Affymetrix Human Genome U133A (HG-U133A) microarrays, which included 22,283 probesets. In light of the data analysis protocol in MicroArray Quality Control (MAQC)-II Project [9], the gene expression data generated from 130 out of 230 clinical samples of breast cancer patients were used as training set and the rest of the 100 cases were used as independent test set. The response to preoperative chemotherapy, which was divided into two subcategories of no residual invasive cancer in the breast or lymph nodes (pCR) and residual invasive cancer (RD), was used as the clinical endpoint for prediction [4].

The acute myeloma leukemia data set included the gene expression profiling of the clinical samples of 242 patients with cytogenetically normal acute myeloid leukemia (CN-AML) [39]. In the training set, the gene expression data of 163 clinical samples were generated by using Affymetrix Human Genome U133A&B (HG-U133A&B) microarrays, which included a total of 44,760 probesets. The gene expression data of 79 clinical samples in the independent test set were generated by using HG-U133Plus2 microarrays. During the calculation procedure, we only used 44,693 common probesets between HG-U133A&B chips and HG-U133Plus2 chips for the DEG selection and predictive model construction. The clinical endpoint of overall survival times was dichotomized with a “milestone” cutoff because the continuous endpoint values cannot be predicted by the binary classification models. By considering the balance between the number of positive samples and that of negative samples, the patients with the survival time less than one year were categorized into the “high-risk” group and the rest with the survival time equal to or longer than one year were assigned to the “low-risk” group. In addition, a patient was excluded from the data set if the survival time was less than one-year milestone cutoff and censored when he/she was still alive. Eventually, there were 152 patients in the training set and 77 patients in the independent test set.

The gene expression data in the glioblastoma data set [7] were generated by using HG-U133A microarrays. In glioblastoma research, a subcategory of glioblastoma termed ProNeural (PN) was highly related to better survival prognosis when compared to other subcategories [6]. In our study, we collected 50 patients belonging to the PN subcategory with a mean survival of 924 days and 50 patients belonging to non-PN subcategory with a mean survival of 150 days. Among these patients, 60 of them, which included 30 patients in PN subcategory and 30 in non-PN subcategory, were randomly assigned to the training set and the rest were used as the independent test set. A predictive model was constructed to discriminate the PN and non-PN categories based on the microarray gene expression data.

### 2.2. Probesets Mapping

For Affymetrix microarray platforms, a gene may be detected by multiple probesets. Before identifying DEGs, we mapped the multiple probesets to a unique HUGO gene symbol by using the probeset with the highest fold change value between two groups of samples. Accordingly, 22,283 probesets involved in the data sets of breast cancer and glioblastoma were mapped to 11,285 unique genes and 44,693 common probesets involved in the acute myeloma leukemia data set were mapped to 14,892 unique genes, respectively.

### 2.3. Identification of Differentially Expressed Genes

Student's *t*-test, which can assess how significant a gene is differentially expressed in two compared phenotypes, was used in our study for the DEG selection. The *P* value for each of the genes was calculated by *t*-test and directly used for gene filtering without multiple-testing correction. Only the genes with *P* < 0.05 were kept. To ensure the reproducibility of the DEG lists generated by the *t*-test, a fold change ranking is usually applied to refining the genes with *P* < 0.05. These genes were ranked by their fold changes (the expression intensity of a gene in sample A/its expression intensity in sample B). Only the genes with fold change >1.5 (FC > 1.5) or fold change <0.667 (FC < 0.667) were kept for the subsequent analysis. Note that, in some microarray studies of clinical samples, only a few genes can meet the fold change cutoff because of the minor phenotypic differences between the two groups of clinical samples.

### 2.4. Construction of Gene-Pathway Bipartite Network

For the purpose of screening out the genes unassociated with the phenotypic differences, we constructed a gene-pathway bipartite network, which can be used to score the genes according to their connections [40] to the cancer-related signaling pathways. All the cancer-related pathways were collected from Kyoto Encyclopedia of Genes and Genomes (KEGG) pathway database and listed in Table 1. The first six pathways reflected the overview of cancers and the rest were correlated with the specific types of cancers.

The bipartite network was a particular class of complex networks, in which the nodes were divided into two groups and the connections only existed between two nodes in different groups [41]. So, the nodes in the gene-pathway bipartite network were genes or pathways and were divided into two groups of gene set and pathway set, respectively. The connections between genes and pathways indicated (1) which genes were involved in a specific pathway and (2) which pathways included a specific gene. We scored each of the genes with a weighting method proposed by Zhou et al. [42]. Let us consider a gene-pathway bipartite network *N*(**G**, **P**, **E**), where *G* and *P* represent the gene set and pathway set, respectively. **E** is the set of connections between genes and pathways. The genes and pathways in **G** and **P** were denoted by *g*_1_, *g*_2_,…, *g*_*n*_ and *p*_1_, *p*_2_,…, *p*_*m*_, respectively. The initial score *s*_0_ assigned to each of the genes in **G** was set to 1. In the first step, we calculated the weights *W*  (*W* = {*w*_1_, *w*_2_,…, *w*_*m*_}) for the pathways via

(1) $$\begin{array}{c} w_{l}=\overset{n}{\underset{j=1}{\sum }}\frac{a_{jl}s_{0}}{k\left(g_{j}\right)}\\ \left(l=1,2,\ldots ,\text{the}\;\text{number}\;\text{of}\;\text{pathways}\;\text{in}\;P\right), \end{array}$$

where *k*(*g*_*j*_) was the degree of the *j*th gene and *a*_*jl*_ was the *n* × *m* adjacent matrix:

(2) $$\begin{array}{c} a_{jl}=\{\begin{array}{cc} 1, & g_{j}p_{l}\in E,\\ 0, & \text{otherwise}. \end{array} \end{array}$$

In the second step, we calculated the final scores *S*  (*S* = {*s*_1_, *s*_2_,…, *s*_*n*_}) for all the genes via

(3) $$\begin{array}{c} s_{i}=\overset{m}{\underset{l=1}{\sum }}\frac{a_{il}w_{l}}{k\left(p_{l}\right)}\;\left(i=1,2,\ldots ,\text{the}\;\text{number}\;\text{of}\;\text{genes}\;\text{in}\;G\right), \end{array}$$

where *k*(*p*_*l*_) and *w*_*l*_ were the degree and the weight of the *l*th pathway, respectively, and *a*_*il*_ was the *n* × *m* adjacent matrix:

(4) $$\begin{array}{c} a_{il}=\{\begin{array}{cc} 1, & g_{i}p_{l}\in E,\\ 0, & \text{otherwise}. \end{array} \end{array}$$

By combining (1) and (3), we can directly calculate the scores for the genes via

(5) si=∑l=1mailk(pl)∑j=1najls0k(gj)=∑j=1ncijs0 (i=1,2,…,the  number  of  genes  in  G),

where

(6) $$\begin{array}{c} c_{ij}=\frac{1}{k\left(g_{j}\right)}\overset{m}{\underset{l=1}{\sum }}\frac{a_{il}a_{jl}}{k\left(p_{l}\right)}. \end{array}$$

The matrix **C** = {*c*_*ij*_}_*n*×*n*_ represented the weighted **G** projection. In our study, the DEGs were firstly selected by the statistical methods and subsequently ranked by the scores **S**  (**S** = {*s*_1_, *s*_2_, …, *s*_*n*_}). Only the DEGs with *s* ≥ 1 were kept as features for the construction of the predictive models.

### 2.5. Model Construction for Clinical Endpoints Prediction

The binary classification models for predicting the clinical endpoints were constructed by using support vector machine (SVM), which is a popular learning machine based on statistical learning theory [43, 44]. In our study, radial basic function was used as the kernel function in SVM. The regularization parameter *c* and the kernel width parameter *σ* were optimized by a grid search approach. For each of the clinical endpoints, the SVM model was built by using the training set and leave-one-out cross-validation and validated by the independent test set. Four performance metrics, namely, specificity, sensitivity, accuracy, and Matthew's correlation coefficient (MCC), were considered for model evaluation and defined as follows:

(7) Specificity=TNFP+TN,Sensitivity=TPTP+FN,Accuracy=TP+TNTP+FP+TN+FN,MCC=(TP×TN−FP×FN) ×((TP+FP)×(TP+FN) ×(TN+FP)×(TN+FN))−1/2,

where TP, FP, TN, and FN represent true positive, false positive, true negative, and false negative, respectively. In addition, the areas under the ROC curves (AUCs) were also provided for evaluating the performance of the models on the prediction of the survival milestone outcomes of AML patients and the molecular subclasses of high-grade glioblastoma. The software libsvm 3.17 [45] used in our study for SVM modeling can be freely downloaded from the website http://www.csie.ntu.edu.tw/~cjlin/libsvm/.

## 3. Results

### 3.1. Model Performance on Predicting the Reoperative Treatment Response of Breast Cancer

**Figure 1:**
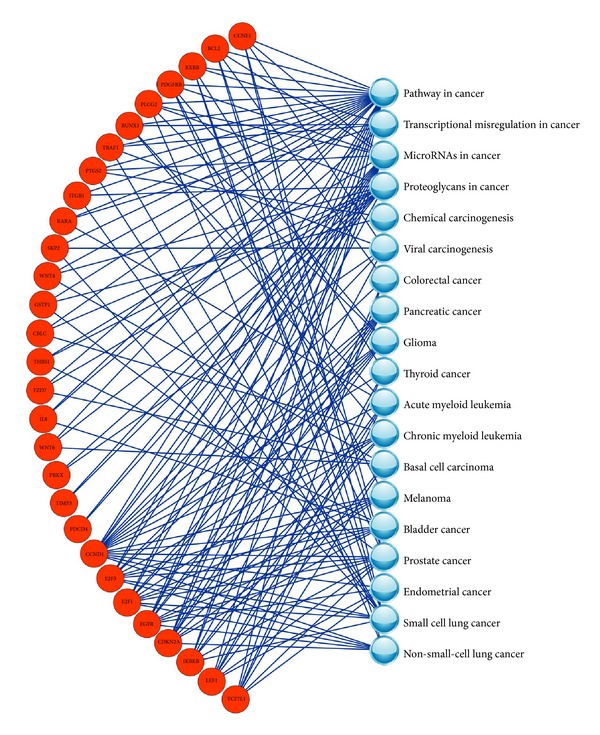
The gene-pathway bipartite network constructed with 29 gene signatures that were used for predicting the reoperative treatment response of breast cancer.

According to the data analysis protocol in MAQC-II project, 130 clinical samples of breast cancer patients were assigned to training set and the rest of the 100 clinical samples were used as independent test set. By comparing the gene expression profiles of the samples in pCR subcategory with those in RD subcategory in training set, 1010 genes with *P* value < 0.05 and |log⁡_2_⁡FC| > 0.585 were selected as DEGs and used to construct the gene-pathway bipartite network. Based on the connections between the DEGs and the cancer-related KEGG pathways, 1010 DEGs were scored by a weighted method and then 29 DEGs with the scores ≥1 were kept as features for model construction. The gene-pathway bipartite network, which connected the 29 DEGs with the 20 cancer-related KEGG pathways, was shown in Figure 1. It can be seen from Figure 1 that the gene* CCND1*, which was ranked 1st in the 1010 DEGs, had the most connections to the cancer-related pathways, indicating it was an important feature for the prediction of the clinical endpoint of breast cancer.

A SVM model was constructed by using the training set and leave-one-out cross-validation. The best parameters of *c* and *σ* were 2 and 0.03125, respectively. The prediction results of training set and independent test set were listed in Table 2. In swap analysis, we repeated the model construction and validation process by training the models with the original independent test set and validating them using the original training set with the same 29 DEGs identified in the original analysis. Meanwhile, the prediction results achieved by MAQC-II candidate models were also listed in Table 2. Compared with the MAQC-II candidate models, our model was more robust and superior in predicting the breast cancer outcomes.

### 3.2. Model Performance on Predicting the Overall Survival Milestone Outcome of Acute Myeloma Leukemia

**Figure 2:**
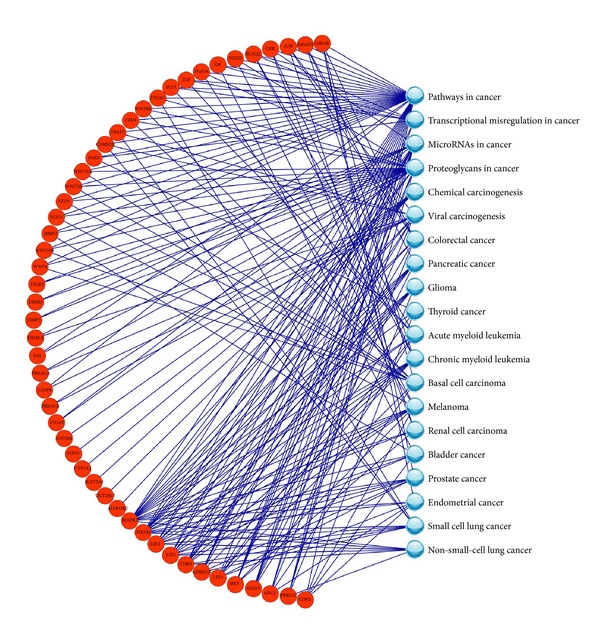
The gene-pathway bipartite network constructed with 50 gene signatures that were used for predicting the overall survival milestone outcome of acute myeloma leukemia.

By comparing the gene expression profiles of the clinical samples between the high-risk patients and the low-risk patients in the training set, 3234 genes with *P* value < 0.05 were selected as DEGs. These DEGs were used to construct the gene-pathway bipartite network and ranked by their scores calculated by the weighted method. At last, 50 DEGs with the scores ≥1 were used as features for the subsequent model construction. The gene-pathway bipartite network of 50 DEGs connected to the 20 cancer-related KEGG pathways was shown in Figure 2. The gene* MAPK1* was ranked 1st in the DEG list and had the most connections to the cancer-related pathways.

In both original and swap analyses, the best parameters of *c* and *σ* optimized for SVM models were 512 and 0.00195, respectively. The prediction results achieved by our models were listed in Table 3. For the convenience of comparison, we built the SVM models in the original and swap analyses with the expression signatures of 86 probesets proposed by the data contributors [39] and summarized the prediction results (Table 3). In general, our model performed similarly to the 86-probeset model in the original analysis, while the MCC achieved by our model with the validation set in the swap analysis was 0.308, which was higher than that achieved by the 86-probeset model.

### 3.3. Model Performance on Predicting the Molecular Subclasses of High-Grade Glioblastoma

**Figure 3:**
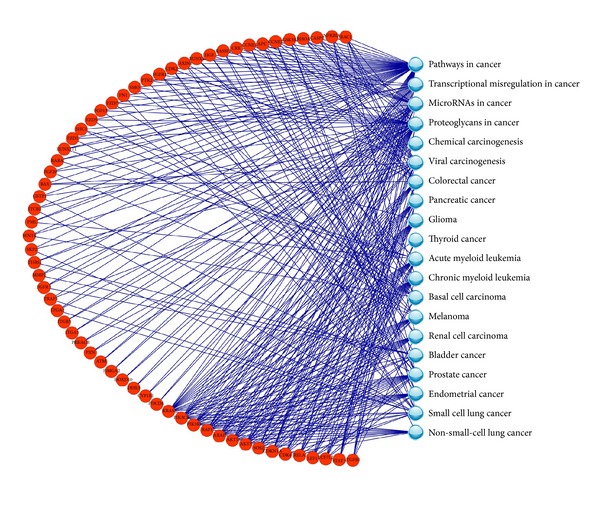
The gene-pathway bipartite network constructed with 62 gene signatures that were used for predicting the molecular subclasses of high-grade glioblastoma.

For the high-grade glioblastoma data set, 2712 genes with *P* value < 0.05 were selected as DEGs and 62 of them with scores ≥1 were used to construct the SVM models. The gene-pathway bipartite network of 62 DEGs connected to the 20 cancer-related KEGG pathways was shown in Figure 3. The gene* KRAS* was ranked 1st in the DEG list and had the most connections to the cancer-related pathways. The best parameters of *c* and *σ* optimized for SVM models in original analysis were 8 and 0.00781, respectively, and were 0.5 and 0.125 in swap analysis, respectively. The prediction results in original and swap analyses were listed in Table 4. In the original analysis, the prediction accuracy for the validation set was as high as 87.5% and similar to that achieved in the training procedure, indicating the superior performance of the SVM model in predicting the molecular subclasses of high-grade glioblastoma. In the swap analysis, the MCC for validation set was dropped to 0.377. This was mainly because the number of samples for the model construction was limited.

## 4. Discussion

In clinical researches, the microarray-based gene expression profiling is often used to construct the models for predicting cancer prognosis. Identifying the DEGs accurately plays a key role in the procedure of clinical model construction. Current statistical methods and machine learning algorithms used for DEG selection only focus on the changes of the gene expression levels between the two groups of clinical samples instead of the causes behind these changes and subsequently result in a number of false positive genes unrelated to the phenotypic differences involved in the DEG list and the predictive models becoming unreliable. In our current study, we described a weighted method, which scored each of the genes according to their connections to the cancer-related pathways in the gene-pathway bipartite network, for the purpose of refining the DEG list generated by the statistical methods. By considering the two facts of (1) how many genes connected to a specific pathway and (2) how many pathways involved a specific gene, all the DEGs in the bipartite network were scored by the weighted method. The DEGs with scores ≥1 were considered as the specific cancer-related genes and used to construct the predictive models.

In order to validate the performance of the predictive models, the gene expression data of the clinical samples were collected in our study to predict three clinical endpoints, namely, the reoperative treatment response of breast cancer, the overall survival milestone outcome of acute myeloma leukemia, and the molecular subclasses of high-grade glioblastoma. For the prediction of reoperative treatment response of breast cancer, 29 DEGs were selected from the bipartite network as features to construct SVM models. In both original and swap analyses, our model performed (MCC = 0.332 and 0.343, resp.) better than the MAQC-II candidate model (MCC = 0.301 and 0.267, resp.). Moreover, in the swap analysis, the MCC achieved by our model in training procedure (MCC = 0.343) was equal to that achieved in validation procedure, indicating the robust model performance. When predicting the overall survival milestone outcome of acute myeloma leukemia, the performance of our model with 50 DEGs was similar to that of the 86-probeset model in original analysis. In the swap analysis, the MCC achieved by our model (MCC = 0.308) was higher than that (MCC = 0.236) achieved by the 86-probeset model. As to the prediction of the molecular subclasses of high-grade glioblastoma, 62 DEGs were used for SVM model construction. The accuracy achieved in the original analysis was as high as 87.5%. Meanwhile, the model performance was robust in the original analysis (MCC = 0.800 and 0.775 in training and validation procedures, resp.). Note that, in the swap analysis, the MCC for validation set was only 0.377. This was mainly because the number of samples used for model construction was limited. In the swap analysis, only 40 samples were used to construct the predictive model, which was insufficient to ensure the reliability of the predictive model.

## 5. Conclusions

In this study, we suggested a strategy to identify the gene signatures, which not only were differentially expressed between two groups of clinical samples but also highly correlated with a specific cancer, from a gene-pathway bipartite network. The predictive models constructed with these gene signatures performed better than those models reported in previous studies. Moreover, in both original and swap analyses, our models achieved similar prediction results, indicating the robust model performance on predicting the cancer prognosis.

## Figures and Tables

**Table 1:** The 20 cancer-related signaling pathways collected from KEGG database for the construction of gene-pathway bipartite network.

Pathway entry	KEGG pathway name	Number of genes
hsa05200	Pathways in cancer	327
hsa05202	Transcriptional misregulation in cancer	179
hsa05203	Viral carcinogenesis	206
hsa05204	Chemical carcinogenesis	80
hsa05205	Proteoglycans in cancer	225
hsa05206	MicroRNAs in cancer	296
hsa05210	Colorectal cancer	62
hsa05211	Renal cell carcinoma	66
hsa05212	Pancreatic cancer	66
hsa05213	Endometrial cancer	52
hsa05214	Glioma	65
hsa05215	Prostate cancer	89
hsa05216	Thyroid cancer	29
hsa05217	Basal cell carcinoma	55
hsa05218	Melanoma	71
hsa05219	Bladder cancer	38
hsa05220	Chronic myeloid leukemia	73
hsa05221	Acute myeloid leukemia	57
hsa05222	Small cell lung cancer	86
hsa05223	Non-small-cell lung cancer	56

**Table 2:** The results of predicting the reoperative treatment response of breast cancer in original and swap analyses.

		Our model				MAQC-II candidate model			
		SP	SE	ACC	MCC	SP	SE	ACC	MCC
Original analysis	Training	0.928	0.455	0.808	0.444	0.847	0.569	0.775	0.433
	Validation	0.882	0.467	0.820	0.332	0.729	0.667	0.720	0.301

Swap analysis	Training	0.988	0.200	0.870	0.343	0.899	0.522	0.837	0.454
	Validation	1.000	0.152	0.785	0.343	0.959	0.212	0.769	0.267

In the prediction, pCR was defined as positive sample.

SP, SE, ACC, and MCC represented specificity, sensitivity, accuracy, and Matthew's correlation coefficient, respectively.

**Table 3:** The results of predicting the overall survival milestone outcome of acute myeloma leukemia in original and swap analyses.

		Our model					86-probe-set model				
		SP	SE	ACC	MCC	AUC	SP	SE	ACC	MCC	AUC
Original analysis	Training	0.697	0.837	0.776	0.542	0.776	0.758	0.733	0.743	0.486	0.746
	Validation	0.574	0.600	0.584	0.170	0.587	0.362	0.800	0.532	0.172	0.581

Swap analysis	Training	0.830	0.700	0.779	0.533	0.765	1.000	0.433	0.779	0.564	0.717
	Validation	0.545	0.756	0.664	0.308	0.655	0.712	0.523	0.605	0.236	0.618

In the prediction, high-risk patient was defined as positive sample.

AUC represented the area under the ROC curve.

See notes under Table 2 for more information.

**Table 4:** The results of predicting the molecular subclasses of high-grade glioblastoma in original and swap analyses.

		Our model				
		SP	SE	ACC	MCC	AUC
Original analysis	Training	0.900	0.900	0.900	0.800	0.900
	Validation	0.750	1.000	0.875	0.775	0.875

Swap analysis	Training	0.950	0.900	0.925	0.851	0.925
	Validation	0.800	0.567	0.683	0.377	0.684

In the prediction, the gene expression profile termed ProNeural (PN) was defined as positive sample.

AUC represented the area under the ROC curve.

See notes under Table 2 for more information.
